# Influence of the Type of Delivery, Use of Oxytocin, and Maternal Age on *POU5F1* Gene Expression in Stem Cells Derived from Wharton's Jelly within the Umbilical Cord

**DOI:** 10.1155/2019/1027106

**Published:** 2019-12-14

**Authors:** Paulina Gil-Kulik, Piotr Chomik, Arkadiusz Krzyżanowski, Elżbieta Radzikowska-Büchner, Ryszard Maciejewski, Anna Kwaśniewska, Mansur Rahnama, Janusz Kocki

**Affiliations:** ^1^Department of Clinical Genetics, Medical University of Lublin, Poland; ^2^Department of Obstetrics and Pathology of Pregnancy, Medical University of Lublin, Poland; ^3^Department of Plastic Surgery, Saint Elizabeth's Hospital, Warsaw, Poland; ^4^Department of Normal Anatomy, Medical University of Lublin, Poland; ^5^Chair and Department of Dental Surgery, Medical University of Lublin, Poland

## Abstract

The paper presents an evaluation of the *POU5F1* gene expression in mesenchymal stem cells derived from Wharton's jelly within the umbilical cord, collected from 36 patients during labor. The study is the first one to show that the expression of *POU5F1* in mesenchymal stem cells has been dependent on maternal age, birth order, route of delivery, and use of oxytocin. Our research proves that the *POU5F1* gene expression in mesenchymal stem cells decreases with each subsequent pregnancy and delivery. Wharton's jelly stem cells obtained from younger women and during their first delivery, as well as patients treated with oxytocin, show higher *POU5F1* gene expression when compared with the subsequent deliveries. This leads to a conclusion that they are characterized by a lower level of differentiation, which in turn results in their greater plasticity and greater proliferative potential. Probably, they are also clinically more useful.

## 1. Introduction

During embryogenesis in mammals, early embryonic cells progressively differentiate from the pluripotent state to separate cell lines. At the same time, they are gradually losing their development potential. Pluripotency, which is characteristic of cells in the inner cell mass (ICM) of a preimplanted blastocyst, is defined as the cell's ability to differentiate into all types of body cells. The pluripotency state is transient in vivo; however, embryonic stem cells (ESCs) derived from the ICM of a blastocyst may possibly retain pluripotency indefinitely in vitro. It has been shown that pluripotency is controlled by a vast transcription network. The *POU5F1* (POU domain, class 5, transcription factor 1) gene is a key component of the pluripotency regulation network, and its interaction has proven to be a determinant of the self-renewal of ESCs or their differentiation into trophoblasts [1]. The *POU5F1* gene is of key importance for the mechanisms governing pluripotency, and it is strongly expressed in pluripotent cells and is silenced after differentiation. Interestingly, as demonstrated by Guilai and Ying [2] and Niwa et al. [3], embryonic stem cells are dependent on the exact level of *POU5F1* gene expression [2, 3].

The OCT-3/4 protein (octamer-binding transcription factor-3/4) encoded by the *POU5F1* gene is more recently treated as an important surface marker for the subpopulation of undifferentiated cancer-initiating cells [4, 5]. The OCT4A protein was found, in close cooperation with SOX2 (SRY-related HMG-box gene 2) and NANOG (homeobox transcription factor Nanog), to regulate the transcription and maintenance of ES cell pluripotency by activating gene expression associated with pluripotency and repression of gene expression associated with differentiation. It has a unique role in the development and determination of pluripotency and constitutes the core of the regulatory network that suppresses genes related to differentiation, thus retaining pluripotency cells [6]. It is known that POU5F1 is also a key transcription factor necessary for self-renewal and survival of MSCs (mesenchymal stem cells) [7, 8]. The OCT4A protein also appears to be the most important factor at the transcription level when reprogramming the human somatic cells into iPSCs (induced pluripotent stem cells) [9].

Stem cell research has recently become one of the most prominent and up-to-date fields of science and technology. Researchers are convinced that studies in this area bring promising results that can lead to successful treatment of many diseases. Such an important scientific breakthrough can revolutionize medical practice and improve the quality of life and life expectancy. This is why mothers' knowledge on the possibilities of using stem cells, as well as the possibility of their banking, has been constantly growing.

In recent years, there has been vast interest in topics connected with mesenchymal stem cells. This is mainly due to their fascinating features, including prolonged ex vivo proliferation, multiline potential, and immunomodulatory properties. In this respect, MSCs become attractive candidates in terms of therapeutic applications, especially those obtained from Wharton's jelly. In addition, MSCs derived from Wharton's jelly represent a more primal population than their adult counterparts, opening new perspectives for cellular therapies [10]. In case of health problems, potential use of MSCs isolated from the umbilical cord gives great hope for curing the future disease [11].

To be able to fully exploit the potential of the mesenchymal stem cells of Wharton jelly, we need to know the factors that affect the state of pluripotency and the ability to self-renew these cells. In our work, we were the first to examine how factors related to pregnancy and childbirth affect the expression level of one of the pluripotency genes—*POU5F1*—in mesenchymal stem cells.

The aim of the study was to evaluate the expression of the *POU5F1* gene in stem cells derived from the umbilical cord of Wharton's jelly, depending on the type of delivery, treatment applied, maternal age, and the number of pregnancies and deliveries.

## 2. Material and Methods

The study included 36 patients hospitalized at the Department of Obstetrics and Pathology of Pregnancy, Independent Public Clinical Hospital No. 1, in Lublin during the delivery. The umbilical cord was taken from the patients. The characteristics of the test group are shown in Tables 1 and 2. The study was carried out with the consent of the Local Bioethical Commission.

### 2.1. Methods

#### 2.1.1. Stem Cell Isolation

Mesenchymal stem cells were isolated from Wharton's jelly within the umbilical cords of pregnant women. The isolation was performed by enzymatic digestion using the enzyme collagenase type I (Sigma, USA); then, the stem cells were cultured for 14 days in adherent conditions [12]. After this period, the cultured cells were removed mechanically using a cellular scraper, and then, they were prepared for cytometric analysis and RNA isolation.

#### 2.1.2. Cytometric Analysis

To determine the phenotypic evaluation of newly isolated stem cells and the cultured cells, cytometric analysis was performed. Monoclonal fluorescently labeled mouse anti-human CD90 PC5-labeled by Beckman Coulter and mouse anti-human CD105 PE- labeled by Beckman Coulter were used to assess the phenotype of the cells. The cytometric analysis was performed in accordance with the authors' protocol [12].

#### 2.1.3. Gene Expression

The isolation of cellular RNA was carried out using the Chomczyński and Sacchi methods [13] followed by spectrophotometric measurements of RNA concentrations and reverse transcription reaction. The real-time PCR method was used to analyze the expression of the *POU5F1* gene. Preliminary results obtained by the real-time PCR method were developed using the Life Technologies Expression Suite v1.03. For the *POU5F1* gene expression, the commercially available TaqMan probe was used for the *POU5F1* gene Hs04195369_s1 and for endogenous control *GAPDH* Hs99999905_m1 (Thermo Fisher Scientific). The level of relative expression of the test gene was calculated from the formula RQ = 2^−ΔΔCt^ [14]. Detailed procedures for the isolation of reverse PCR transcription and real-time PCR reactions have been described in our work [15]. The statistical analysis was performed using the Statistica v. 13.0 software. The statistical analysis was performed using the Mann-Whitney *U* test and Spearman's correlation test. Significant results were recognized for *p* < 0.05.

## 3. Results

### 3.1. Cell Culture and Cytometric Analysis

**Figure 1:**
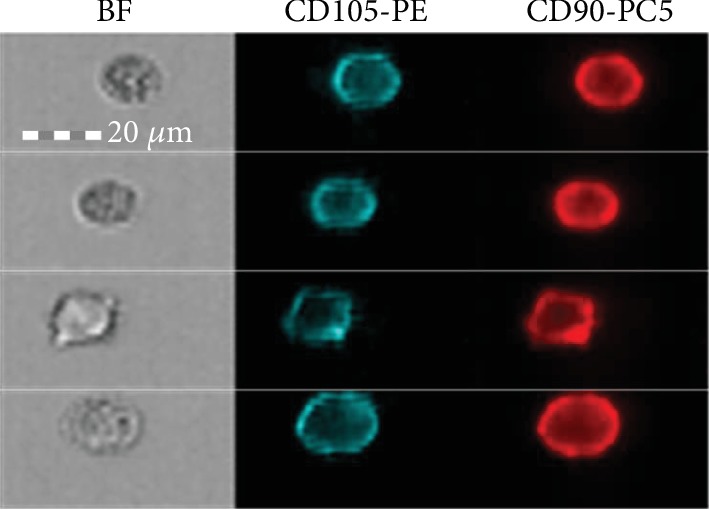
Exemplary stem cells from a 14-day-old cell culture showing a microscopic image in a bright field (BF) and fluorescence in individual channels depicting the expression of CD105 and CD90 antigens. Photographs taken with the Amnis FlowSight flow cytometer.

**Figure 2:**
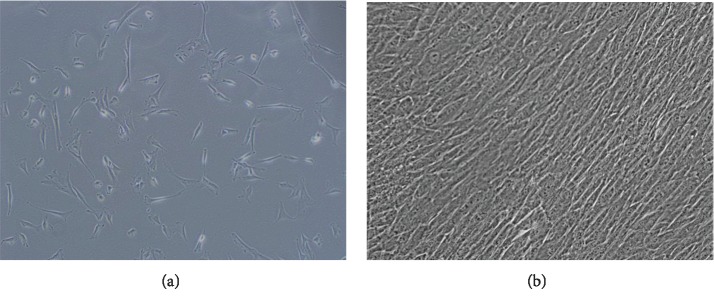
Photographs of exemplary stem cells from (a) a 3-day cell culture and (b) a 14-day cell culture showing a microscopic image in a bright field (BF). Photographs taken at 100x magnification using the Xcellence RT system with an IX81 inverted microscope from Olympus.

The cytometric analysis (Figure 1) and cell culture (Figure 2) confirmed the native character of the analyzed cells. The ability of the cells to adhere to plastic walls and the fibroblast-like shape of the cells have been demonstrated. The cytometric analysis showed the presence of surface antigens in the cells: CD90 (22 ± 8.6%) and CD105 (53 ± 9.8%).

### 3.2. Gene Expression Analysis

The presented study analyzed the level of *POU5F1* gene expression in cells derived from Wharton's jelly within the umbilical cord collected from patients during labor. The *POU5F1* transcript was shown in all the samples tested.

### 3.3. Effects of the Type of Delivery

**Figure 3:**
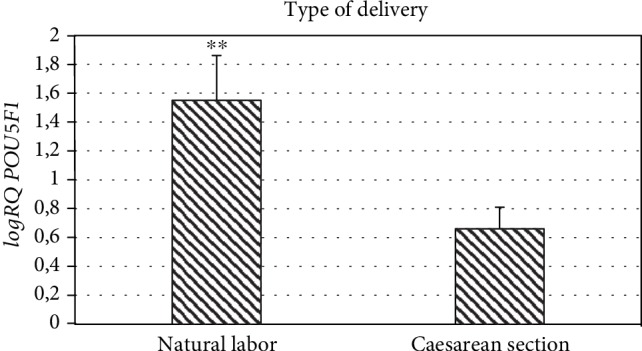
Mean *POU5F1* gene expression (logRQ ± SE) in stem cells derived from the umbilical cord depending on the type of delivery. Mann-Whitney *U* test (^∗∗^*p* < 0.01).

As a result of the conducted studies, it was observed that *POU5F1* expression depends on the type of delivery. Significantly higher mean *POU5F1* gene expression (*p* = 0.008) was demonstrated in patients who had natural birth (logRQ ± SE = 1.55 ± 0.31) compared to women who gave birth by cesarean section (logRQ ± SE = 0.66 ± 0.15) (Figure 3).

### 3.4. Effects of Maternal Age

**Figure 4:**
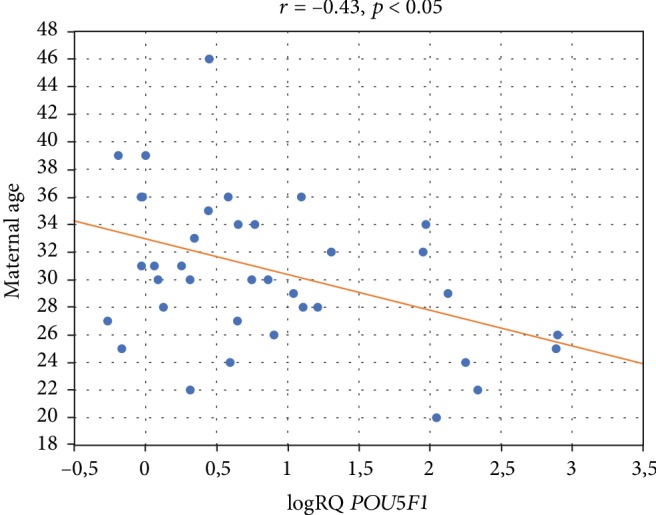
Graph of the *POU5F1* gene expression (logRQ) against maternal age. Spearman's rank (*r* = −0.43, *p* < 0.05).

In addition, the *POU5F1* gene expression in Wharton's jelly cells was shown to be dependent on maternal age. There is a negative correlation of *POU5F1* expression (*r* = −0.43, *p* < 0.05) with maternal age in the examined material, and the analysis proves that the younger the woman is when giving birth, the higher the *POU5F1* expression (Figure 4).

### 3.5. Effects of the Number of Previous Pregnancies and Deliveries

**Figure 5:**
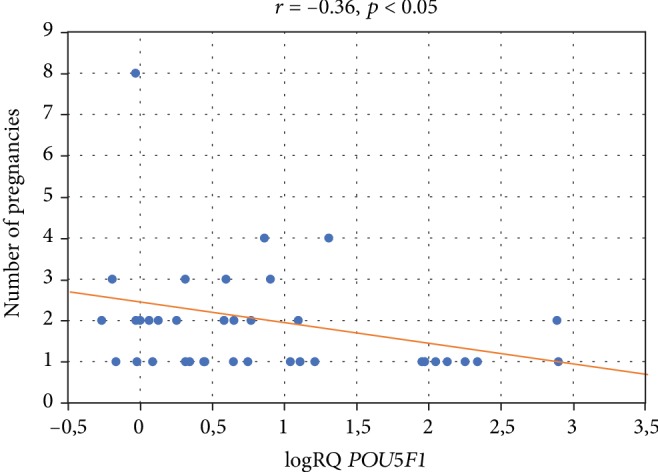
Graph of the *POU5F1* gene expression (logRQ) against the number of pregnancies. Spearman's rank factor (*r* = −0.36, *p* < 0.05).

**Figure 6:**
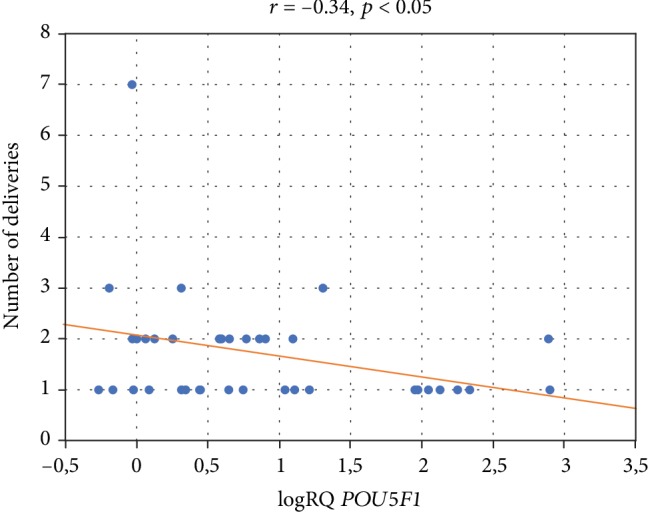
Graph of the *POU5F1* gene expression (logRQ) against the number of deliveries. Spearman's rank factor (*r* = −0.34, *p* < 0.05).

**Figure 7:**
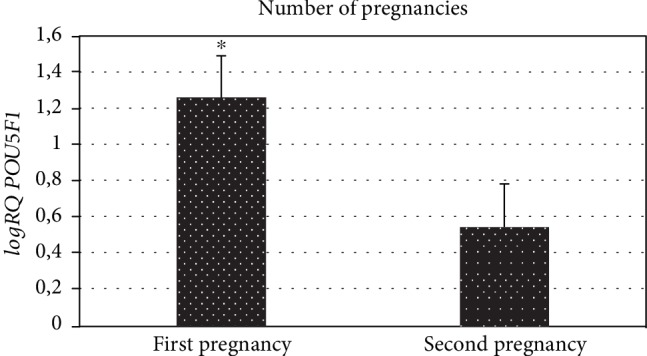
Mean *POU5F1* gene expression (logRQ ± SE) in stem cells derived from the umbilical cord depending on the number of pregnancies. Mann-Whitney *U* test (^∗^*p* < 0.05).

We demonstrated the association of *POU5F1* gene expression with the number of pregnancies and deliveries. There was a negative correlation between the *POU5F1* gene expression and the subsequent pregnancy (*r* = −0.36, *p* < 0.05) (Figure 5) and subsequent deliveries (*r* = −0.34, *p* < 0.05) (Figure 6). It was observed that with each subsequent pregnancy and delivery, the expression of the *POU5F1* gene decreases in the cells of Wharton's jelly within the umbilical cord. In addition, there is significantly higher *POU5F1* gene expression (*p* = 0.03) in patients in the first pregnancy (logRQ ± SE = 1.26 ± 0.23) compared to patients during the second pregnancy (logRQ ± SE = 0.54 ± 0.24) (Figure 7).

### 3.6. Effects of Oxytocin Used

**Figure 8:**
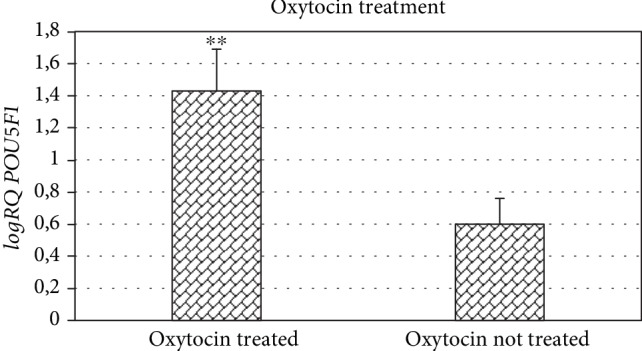
Mean *POU5F1* gene expression (logRQ ± SE) in stem cells derived from the umbilical cord depending on the administration of oxytocin. Mann-Whitney *U* test (^∗∗^*p* < 0.01).

The analysis of the dependence of *POU5F1* gene expression against the treatment used showed significantly higher *POU5F1* expression in Wharton's jelly cells from patients who received oxytocin during labor (Figure 8).

There were no significant differences in the *POU5F1* gene expression in Wharton's jelly cells within the umbilical cord depending on the week of pregnancy, the birth weight of the newborn, and the sex of the newborn.

## 4. Discussion

There are many local, systemic, and environmental factors affecting stem cells [16], and data coming from the literature show that the performance of stem cell functions changes during aging [17]. Many previous studies have touched upon the topic of the effects of aging on the regenerative capacity of living organisms and their effect on biological activity [18]; however, only some have investigated the impact of the age of the mother on stem cells derived from the umbilical cord.

Our own studies carried out on 36 umbilical cords taken from women in labor aged 20-46 showed a negative correlation of the *POU5F1* gene expression in stem cells derived from the umbilical cord with the patient's age. Our results correspond with the results obtained by Alrefaei et al. [19]. The authors showed a negative correlation of CD105 and CD29 immunoexpression in different parts of the umbilical cord with the mother's age, as well as a reduced amount of MSCs in older mothers [19].

No previous studies have investigated the influence of maternal age on the expression of a key transcription factor necessary for the self-renewal and survival of the MSC—the *POU5F1* gene. Our reduced *POU5F1* gene expression in older patients suggests that isolation and use of MSCs from Wharton's jelly from younger women giving birth will probably provide a more beneficial therapeutic outcome.

Oxytocin is a naturally produced endocrine hormone that has been approved for use in women during labor and is not associated with many harmful side effects. Oxytocin stimulates uterine contractions during labor and stimulates milk production during breastfeeding, and due to its similarity to vasopressin, it may reduce urine output and stimulate sodium secretion. As a neurotransmitter, oxytocin plays a role in social behavior in many species: maternal behavior, sexual arousal, increased confidence, and reduced anxiety; it also has antistress functions: for example, it reduces blood pressure and cortisol levels [20–22]. Oxytocin receptors are present during early development in many tissues among other neural progenitor cells suggesting that oxytocin is intricately involved in modulating facets of early development [23]. Oxytocin seems to be essential for optimal skeletal remodeling [24]. It is also essential for the proper regeneration of muscle tissue and homeostasis, and plasma oxytocin decreases with age. Inhibition of oxytocin signaling in young animals reduces muscle regeneration, while systemic administration of oxytocin rapidly improves muscle regeneration by increasing the activation and proliferation of old muscle stem cells by activating the MAPK/ERK signaling pathway [25–27]. In addition, according to Elabd et al., a genetic lack of oxytocin does not cause muscle malformations but leads to premature sarcopenia [25]. Decreased levels of circulating oxytocin are associated with pathological conditions, such as autism in children [28], osteoporosis [29], or depression [30]. Oxytocin has also been tested to improve the psychological wellbeing of the elderly and is the subject of clinical trials in the treatment of autism, schizophrenia, and depression [31, 32].

Our own studies presented in this study showed that in the group of patients receiving oxytocin, the *POU5F1* gene expression in umbilical cord stem cells is statistically significantly higher compared to the expression in stem cells from women in whom oxytocin was not used.

Mauro et al., in their studies, by means of immunohistochemistry and RT-PCR, showed the expression of oxytocin protein in the umbilical cord mainly in the mesenchymal cells of Wharton's jelly and in the vascular endothelium [33]. Yong Sook et al. cultivated mesenchymal stem cells isolated from umbilical cord blood with oxytocin and examined for their therapeutic effect in the heart with myocardial infarction. In rats injected with oxytocin MSC, cardiac fibrosis in the peri-infarct zone decreased. The authors showed that oxytocin is an effective primer for stem cells for use in damaged heart tissue; also, oxytocin stimulates the cardiomyogenesis of embryonic stem cells and endothelial cell proliferation [34]. Jafarzadeh et al. demonstrated that oxytocin treatment can promote neural differentiation of the adipose tissue-derived stem cells [35].

Cattaneo et al. show that oxytocin has the ability to stimulate human umbilical vein endothelial cell motility and invasion. The authors suggest that oxytocin can participate in processes like angiogenesis where activation of endothelial cells plays a crucial role [36]. Elabd et al. presented evidence that oxytocin may play a major role in the balance of osteoblasts and adipocytes from MSC as well as in the control of the fate of mesenchymal stem cells [37] and thus may affect the *POU5F1* gene expression in mesenchymal cells in Wharton's jelly. de Jamblinne et al. show that several obstetric parameters have influence on the quality of the cord blood unit, like cord clamping, a more advanced gestational age, higher birth weight and higher weight of the placenta, and also the use of oxytocin during labor [38].

Oxytocin is used in regenerative medicine because of its beneficial characteristics, which improve vascular and metabolic functions and are shown to be cardioprotective in obesity and diabetes [22, 26, 39, 40]. Studies show that oxytocin has anti-inflammatory activity, increases stem cell resistance to oxidative stress, and promotes migration, proliferation, and differentiation of mesenchymal stem cells [39, 41–46]. The use of oxytocin together with stem cells has a regulatory effect on growth and differentiation [47–52]. Kim et al. show that injection with oxytocin-treated mesenchymal stem cells from the umbilical cord into the rat heart after ischemia-reperfusion injury improves the engraftment rate and results in an enhanced cardioprotective effect. The authors also speculate that oxytocin can be used as an efficient priming reagent for improving therapeutic potential in cell transplantation therapy [34]. Noiseux et al. speculate that preconditioning of mesenchymal stem cells by oxytocin can be used to improve their therapeutic effect [34]. Kim et al. also show the use of oxytocin as a priming reagent in stem cell therapy because of its ability to restore angiogenic capacity of diabetes-insulted mesenchymal stem cells [53].

In spite of numerous reports regarding the function of oxytocin in the body or the potential effects on homeostasis and repair of tissue, no previous studies revealed the effect of oxytocin administered to pregnant women on *POU5F1* gene expression in Wharton's mesenchymal cells. We speculate that the positive effect of oxytocin on cell proliferation and differentiation is due to its effect on *POU5F1* gene expression.

To date, there have been no reports in the literature about the differences between caesarean section and natural delivery in the context of the *POU5F1* gene expression in MSC cells isolated from Wharton's jelly. Our research has shown that in the group of patients who gave birth by forces of nature, *POU5F1* gene expression in stem cells from the umbilical cord is significantly higher compared to the expression in stem cells from women who gave birth via caesarean section. Perhaps increased expression of *POU5F1* in women giving birth naturally can be due to the effect of endogenic oxytocin, which is physiologically secreted during delivery by natural forces, but this requires confirmation in further research; in this study, we have not tested the level of endogenous oxytocin.

According to Mazzoccoli et al., there are several studies on the role of maternal and neonatal factors associated with the umbilical cord blood potential including gestational age, sex of the newborn and birthweight, placenta mass, and mother's working time and delivery. These are factors that have a significant impact on the umbilical cord blood volume or the number of stem cells [54].

The studies of Thame et al. have shown that the order of births has a significant effect on the number of stem cells—the firstborn child had a higher total number of nucleated cells expressing the CD34 + CD45 + surface antigen and a greater number of nucleated red blood cells (NRBC) in umbilical cord blood [55].

Our studies show that there is a negative correlation between the *POU5F1* gene expression and the number of deliveries; in each subsequent labor, the *POU5F1* gene expression decreases. Moreover, we have shown that in the group of patients who were pregnant for the first time, the *POU5F1* gene expression in umbilical cord stem cells is statistically significantly higher compared to the expression in stem cells from women who were pregnant for the second time. Perhaps, the woman body during the first delivery or pregnancy requires greater adaptation, which is commensurate with higher *POU5F1* gene expression or more stem cells. Lowering the expression of the *POU5F1* gene in subsequent pregnancies may also be related to the decrease in *POU5F1* expressed in our experiment, with the age of the woman in labor.

The state of pluripotency of stem cells is determined by among others a high level of expression of transcription factors such as POU5F1. In our study, we showed a higher expression of the *POU5F1* gene in Wharton's jelly stem cells in younger women, during the first delivery, by the forces of nature, and in patients in whom oxytocin was used. Increased *POU5F1* gene expression in these cases can be associated with a lower degree of differentiation of these cells, greater plasticity, and the ability to self-renew which probably make them clinically more useful, but it must be confirmed by further research.

## 5. Conclusions

The research performed has been the first to show that the expression of *POU5F1* in the mesenchymal stem cells of Wharton's jelly obtained from the umbilical cord depends on the age of the pregnant woman, the route of delivery, and the use of oxytocin. Our research proves that the expression of *POU5F1* in mesenchymal stem cells decreases with each subsequent pregnancy and each subsequent delivery. Wharton's jelly stem cells obtained from younger women and during the first delivery and from patients in whom oxytocin was used show a higher expression of *POU5F1* in comparison to subsequent deliveries, which can be considered to be characterized by a lower level of differentiation, which results in greater plasticity and greater proliferative potential and is probably clinically more useful. The demonstrated effect of the studied factors on the *POU5F1* gene expression, which is a key factor in stem cell pluripotency, may be important in making a decision regarding cord blood banking or MSCs isolated from the umbilical cord.

## Figures and Tables

**Table 1:** Characterization of the age of the pregnant woman, week of pregnancy, and birth weight of the newborn baby.

Parameter	*N*	Mean	Median	SD	SE
Maternal age (years)	36	30.50	30.00	5.15	0.86
Week of pregnancy		39.00	40.00	2.52	0.42
Newborn weight (g)		3329.17	3430.00	675.92	112.65

**Table 2:** Numbers in individual subgroups, depending on the number of pregnancies and deliveries, sex of the newborn, the type of delivery, and oxytocin treatment.

Parameter	*N*	%
Number of pregnancies		
1	17	47
2	12	33
3	4	11
4	2	6
8	1	3
Number of deliveries		
1	18	50
2	14	39
3	3	8
7	1	3
Gender of the newborn baby		
F	16	44
M	20	56
Type of delivery		
Natural labor	9	25
Caesarean section	27	75
Oxytocin use		
Treated	12	33
Not treated	24	67

## Data Availability

The data used to support the findings of this study are included within the article and can be available from the corresponding author.
